# The Effectiveness of Theory of Mind Training On the Social Skills of Children with High Functioning Autism Spectrum Disorders

**Published:** 2015

**Authors:** Narges ADIBSERESHKI, Abbas NESAYAN, Roghayeh ASADI GANDOMANI, Masood KARIMLOU

**Affiliations:** 1 Department of Psychology and Education of Exceptional Children, Faculty of University of Social Welfare and rehabilitation Sciences, & pediatric Neurorehabilitation Research Center. Tehran, Iran; 2 PhD in Psychology & Education of Exceptional Children, Department of Psychology, University of Bojnord, Bojnord, Iran; 3 Department of Statistics, University of Social Welfare and Rehabilitation Sciences, Tehran, Iran

**Keywords:** Theory of Mind, Children, High Functional Autism Spectrum Disorders, Social skills

## Abstract

**Objective:**

Children with Autism Spectrum Disorders (ASD) tend to have problems in establishing and maintaining their social relationships. Some professionals believe this social impairment is the result of deficit in Theory of Mind (ToM). This study was conducted to explore the effectiveness of ToM training on such children.

**Materials & Methods:**

A quasi-experimental method, pre- test, post-test with control group was used. The sample included of 12 girls and 12 boys with High Functioning Autism Spectrum Disorders (HFASD). Two instruments were used as follows: the Theory of Mind test and the social skills questionnaire (1). The samples were randomly placed in the experimental and control groups. The experimental groups had 15 sessions of ToM training and the control groups had just regular school program.

**Results:**

The data were analyzed by Kolmogorov-Smirnov, independent t- and twoway- variance tests. The scores for social skills in the experimental group were significantly more than the control group.

**Conclusion:**

ToM training might improve the social skills of children with autism spectrum disorders.

## Introduction

Autism is recognized as a neuropsychiatric disorder that affects verbal and nonverbal communication and social skills of autistic individuals. According to The American Psychiatric Association (2000), autism is characterized by impairments in social skills and communication, and the presence of stereotyped/ repetitive behaviors. The main deficits in Autism Spectrum Disorders (ASD) are abnormalities in social behaviors (2), which cause further difficulties. As indicated earlier (3-6), such behaviors can affect learning essential for school, as well as the social skills of individual. In regards to social skills, differing definitions are offered. Stella et al. (7) describe social behavior in the context of orientation and communication, and Wing et al. (8) indicate it in terms of interactions, play, and communication. Laushey and Heflin (9) consider skills such as getting the attention of another, waiting for his/her turn and asking for objects. Learning Theory conceptualizes social skills as discreet, observable responses essential for a child to adapt to and cope with his/her environment. Typically, the rationale for a child with ASD is to fail to perform these skills and creates a deficit in knowledge about how a person should possess and display behaviors. As a result, identifying the deficits in social skills and then remediating them, via operant and/or social learning procedures would be important (10). Developmental model recognizes ASD as a neuro developmental disorder, which are pervasive across all domains of behavior, including social skills (11). Some social skills that are problematic in autistics are smiling at people (whom he/she knows), initiating conversation, saying thank you, call the people by their name, and exhibits eye contact (12). It seems that common themes are a set of observable behaviors, which come into two general categories of verbal and nonverbal communication (13). Children with autism, besides having poor conversational skills (14), may also lack ToM (15). Individuals with autism are impaired in ToM; the ability to understand mental states such as thoughts, intentions and beliefs that influence human behavior (16). ToM is about the mind and how it is needed for all human interactions, such as understanding, explaining, predicting, and manipulating the behavior of others. ToM is required for perceiving social environment and its involvement is necessary in competitive social behaviors (17). Deficiency of ToM in children can cause them to be rejected by their peers (18-23). The findings of studies about ToM development and its effect on the social relationships in different stages (infancy to school age) support the claim that development in ToM can transform children’s relationships (24). Focusing on infants, Spelke et al. (25) describe those infants from the age of 6 months who can recognize animate agents are self-propelled and know biological and mechanical movements (26). This can help them to attend selectively to human behavior and see the events from the agents’ viewpoint. Later in life, toddlers like to play and pretend play. Pretend play motivates them to initiate and sustain social contacts that facilitate cooperative interaction (27, 28). Interactions, especially with siblings, have a powerful influence on children’s socio-emotional adjustment (29). From a ToM perspective, another area, which develops in this stage, is emotional regulation that could influence the child’s relations and its deficits can cause disruptive behavior disorders in children (30). In addition, in this stage, internal language is the most important ability, which toddlers acquire (31, 32). Language development helps them to know other’s feeling and desire (33) and enhances their communication skills and interactions. In preschool years, children understand mental states, especially emotions associated with empathy (34), positive peer interactions (35) and the use of rules for controlling emotional displays (36). Most children can attribute mistaken beliefs to themselves and others and establish advanced social interactions. The development in ToM is associated with how children interact socially and whom they engage with (28). During school, children’s knowledge about mental representations continues to increase and development of ToM increases the social harmony. There is evidence suggesting that some social cognitive skills for understanding false belief could be related to social competence. Children with autism who are deficient in social skills have problems with understanding false belief tasks (37-39), and children with higher understanding of these tasks can make friends and better participate in the activities with them (40). Intervention programs are established to improve ToM and social skills in children with ASD (41). Children who had been trained showed better false belief performance on ToM tasks (42). Sagger (43) studied the effectiveness of ToM training as part of social skills training program for students with autism and the results indicated that the social skills and ToM improved in these students. The results of a study on the effects of ToM training and social skills training indicated a substantial increase in social interactions and ToM scores (44). The outcome of Gever et al. study (43) on ToM-based social cognition training program for school-aged children with pervasive developmental disorders, showed significant progress in all socialization sub domains: interpersonal relationships, play/leisure and social skills. Given the variation in the nature of treatment programs, the effectiveness of intervention programs are limited and inclusive (45) but these programs are used widely (46, 47). The purpose of this study was to examine the effectiveness of ToM training on the social skills of children with high functionality of ASD. 

## Materials & Methods

**Participants**

A quasi-experimental method with pre-test and post-test was used for this study. Our sample included 24 children (7-12 yr old) with High Functioning Autism Spectrum Disorders (HFASD) that randomly were placed in two groups (12 in the experimental and 12 in the control group). These students had to meet some criteria for entering the study. These criteria were student’s scores in the theory of mind test, which must have been 19 or less, making sure that they were not in any risk participating in this study, and informed consent taken from their parents. Pupil’s parents provided informed consent for their child’s participation in the study.

**Materials**

1- A 38-item ToM test, the Wechsler Intelligence Scale for children Revised (WISC-R), and Vineland adaptive behavior scale; -38-item ToM test: the main form of this test was designed by Steerneman (48). This was constructed based on a multidimensional and developmental view, assessing more complex, advanced aspects of TOM, and covering a wider range of ages in comparison with earlier types of the test. This test was administered individually containing images and stories presented to the participant. The questions are given to the subjects. The score of “1” is considered for the correct answer and the score of “0” for the wrong answer. The subjects can earn scores from 0 to 38. A higher score means that the subject ascended to a higher theory of mind level (49). The test also was standardized within Iranian society (49) in a group of students with intellectual disabilities. For validity, the content validity, the correlation of sub-tests with total score, and concurrent validity was used. The correlation coefficient of sub-tests and the total test scores were all significant and varied in the range of 0.82 to 0.96. The reliability was assessed by the means of test-retest and Chronbach’s α. Test-retest varied between 0.70 and 0.94. Internal consistency for total test and each sub-test was estimated at 0.86, 0.72, 0.80, and 0.81 respectively. The reliability coefficient was estimated to be 0.98 (49). 2- Social Skills Rating System (SSRS): This scale is prepared and represented by Gresham and Elliot (1). This scale consists of three separate rating forms; parent, teacher and self-report form, which in this study the teacher and parent forms were used. The SSRS measures factors such as cooperation, assertiveness, self-control in the social skills domain. This scale was standardized in Iran by Shahim (50) and was used in this study.

**Procedure**

Twenty-four children whom had the aforementioned criteria were selected for the study and randomly were placed in experimental and control group (12 in each group). After taking the ToM test, students of score of 19 or below in ToM test, were chosen. In the next step, mothers and teachers filled out the parent and teacher form. After finishing the testing processes, our samples were randomly assigned into two groups: the experimental group and the control group. The experimental group participants were given intervention and the control group participants did not have any intervention, just the regular school program. Intervention program included of a package containing the material and content, which were studied and prepared by the researchers. In addition, it was decided that one of the researchers train two special education students who graduated in this field. After the training, they were asked to follow the instructions and work with a child with ASD (who was not from neither of the schools in the study) in the presence of the researchers. When they got the OK from the trainer, the intervention was started. The intervention was conducted in such a way that each student had an individual instruction 3 times a week for 15 sessions. The trainer instructed 3 students every other day. Every student in the experimental group had the intervention. The intervention sessions consisted of: -Four sessions of instructions about the emotions: Pictures and drawings of cartoon characters with different kinds of emotions like happiness, sadness, fear and aggression were shown to the child who was asked to determine the emotions. In addition, before testing, the examiner had prepared a place for the picture of each emotion on his/her table. After presenting the pictures, the examiner asked the child to put each picture in its place. In the case of a mistake, the correct answer and feedback was given to the child. -Two sessions were run for the instruction of situational emotions. A collection of cartoon pictures, which showed different kinds of situational emotions, was presented to the child. After showing the picture and describing the event in that picture, the child was asked about the emotion of the person in the story. In addition, he/she had to answer all the questions about why the person in the study had such an emotion. If there were any mistakes, feedback would have been given immediately by the examiner. -Three sessions of instructions about desire were also orchestrated. A collection of cartoon pictures was presented to the child. Every story had two pictures. Both the pictures, and the events in them, were described for the child. The child was then asked to talk about the desire of the person in the picture. Meanwhile, the examiner helped the child by pointing to the picture. In the next step, the child was asked about the emotion of the person in the picture. If any mistakes were present, the correct answer and feedback was given to the child. -Three sessions of instruction about beliefs were also taught. A collection of cartoon pictures was presented to the child. Every story had two pictures. Both the pictures, and the events in them, were described for the child while he/she was asked to talk about the belief of the person in the picture. Meanwhile the examiner helped the child by pointing to the picture. In the next step, the child was asked about the emotion of the person in the picture and was asked about why the person in the story had such feelings. If the answer provided had been wrong, the correct answer and feedbacks would have been given to the child. -Five sessions of instructions about desire- beliefs were also instructed: A collection of cartoon pictures were presented to the child. Every story had two pictures. Both pictures and the events in them were described for the child. He/she was then asked to talk about the desire and belief of the person in the picture. Meanwhile the examiner helped the child by pointing to the picture. In the next step, the child was asked about the desire and belief of the person in the picture and was provided the answer about why the person in the story had such feelings. If there had been any mistakes, the correct answer and feedback would have given to the child. After finishing the intervention sessions, post-tests of Social Skills Rating System (SSRS) were performed for the experimental and control groups. The parents and teachers were filled out the forms for the rating. The SPSS ver. 16 (Chicago, IL, USA) was used to analyze the data. By statistical indexes such as mean, mean differences and ANCOVA the data were analyzed.

## Results

Table 1 shows some demographic information for the experimental and control groups. In each group, the number of boys and girls, the mean and standard deviation for the age and IQ of the two groups are presented. The mean age and IQ of the experimental group was M=9.58, M=71.08 and for the control group was M=9.75, M=71.33. These scores show the age and IQ of participants in two groups are close and in the same range. Some descriptive statistics for the social skills scores of two groups (experimental and control) in pre-test and post-test situations (from the parent and teacher forms) are shown in Table 2. There was a significant difference between the pre-test and post-test scores in the experimental group regarding parents’ form (M=33.41, M=39.83) and teachers’ forms (23.08, M=29.08), (P<0.001). However, such discovery was not noticed in the control group between their pre-test and post-test. To determine the effect of theory of mind training, the comparison of mean difference of social skills in pre-test, post-tests of two groups (experimental and control), using ANOVA was considered. There was not a significant difference between the two group in social skills (Parent Form, F=147.71, P=0.001). (Table 3) To determine the effect of theory of mind training, the comparison of mean difference of social skills in pretest, post-tests of two groups (experimental and control), ANOVA was used. There was a significant difference between the two group on social skills (Teacher Form, F=296.51, P=0.001).

**Table 1:** Demographic Information of Students in Experimental and Control Group

**Group**	**Experimental Group**			**Control Group**		
Gender	Boys	Girls	Total	Boys	Girls	Total
N (%)	6 (50%)	6 (50%)	12(100%	6 (50%)	6 (50%)	12 (100%)
	M (SD)	M (SD)	M (SD)	M (SD)	M (SD)	M (SD)
Age (yr)	9.66(1.63)	9.83(1.47)	9.74(1.67)	9.50(1.87)	10 (1.41)	9.75 (1.51)
IQ	70.83(.75)	72 (1.26)	71.08(.99)	71.33(1.21)	71.16(1.16)	71.24(1.12)

**Table 2:** Comparison of Mean Differences of Social Skills(Parents & Teacher Forms)

	**Group**	**N**	**Mean**	**Std. Deviation**	**Std. Error Mean**
Pre-test Parent Form	Experimental	12	33.4167	7.35414	2.12296
	control	12	32.8333	6.05780	1.74874
Post-test Parent Form	Experimental	12	39.8333	7.74401	2.23550
	control	12	33.0833	6.28792	1.81516
Pre-test teacher Form	Experimental	12	23.0833	4.16606	1.20264
	control	12	23.3333	4.14144	1.19553
Post-test teacher Form	Experimental	12	29.0833	5.14266	1.48456
control	12	24.0833	4.12219	1.18997

P<0.001

**Table 3:** Comparison of Mean Differences in Pre-Test, Post-Tests of Social Skills (Parent Form)

**Source**	**Type III Sum of Squares**	**df**	**Mean Square**	**F**	**Sig.**	**Partial Eta Squared**
**Pre** **-** **social skills**	1062.405	1	1062.405	693.341	.000	.971
**Group**	226.348	1	226.348	147.718	.000	.876
**Error**	32.178	21	1.532			
**Total**	33269.000	24				

**Table 4:** Comparison of Mean Differences in Pre-Test, Post-Tests of Social Skills (Teacher Form)

**Source**	**Type III Sum of Squares**	**df**	**Mean Square**	**F**	**Sig.**	**Partial Eta Squared**
Pre-Social skills	466.012	1	466.012	827.838	.000	.975
Group	166.916	1	166.916	296.514	.000	.934
Error	11.821	21	.563			
Total	17588.000	24				

## Discussion

The goal of this study was to examine whether ToM training improved the social skills of students with autism spectrum disorders. In this study, the results of Social Skills Rating showed a significant difference between experimental and control groups in which the experimental group, which received the training, had higher scores than control group. The outcome indicated that ToM training led to improvement of social skills in these children. Theory of mind, the ability to make inference about mental state to self and other people, is thought to be a mechanism needed for human to function in the social networks. Individuals with ASD are deficient in ToM abilities. They may not appreciate that behavior is motivated by mental state and if it is true individuals with ASD should be deficient when they use ToM to represent and take part in interactions (51). The comparison of two groups (experimental and control) in our study indicated that the intervention was effective and the social skills of experimental group were enhanced by the training. The results of this study confirm the conclusions of some researches worked on the social skills of individual with autism (52) and some specifically focusing on ToM (51, 53-57). Frith et al. (58) were interested to find out about real life competencies of those children with autism who passed ToM test. They distinguished social behavior in Interactive (require ToM to perform the behavior) and Active (not require ToM and could be learned). There was a significant advantage in Active sociability for children who passed ToM test. Children who passed false belief tasks showed more every day social insights, but less simple sociability than those who failed. In addition, normal 4-year olds who passed false belief tasks were more socially oriented than those who fail. In the study of Begeer et al. (51) on ToM training for children with autism, the treatment was more effective on conceptual abilities than social skills. The understanding of beliefs and false beliefs were improved but no impact on parent reported social behaviors. Trained children with ASD had significant improvement in their performance on ToM tasks, which remained stable in after follow up period (57). Mental state discourse (ToM) is related to the development of social understanding (59-61). This relates to social cognitive approach in that others have minds, which are fundamental to a self-other understanding within relationships (62). Some studies focused on ToM tasks and interactions of children to find out more about their connection. Dunn and Cutting (35) examined the relation between ToM tasks and interaction of preschoolers and their friends. They found that cooperative pretend play and the interactions of children are associated with false belief understanding, affective perspective taking and emotion understanding. There is a relationship between play interactions of friends and false belief understanding (63). The author argued that there was more evidence that ToM predicted the play behavior than the reveres pathway. Our study’s results from teachers view indicated that the training was effective and the social skills were improved for the group who received the intervention (experimental group). Begeer et al. (51) believe that the findings of different researches in this area can depend on the focus of treatment programs and materials used in ToM training. They used extensive belief and false belief materials in their training and these tasks were improved during and after training. In fisher and Happe (57) study, as reported by teachers, the training was not effective on children’s emotional recognition of skills and daily life ToM uses. They state that small sample sizes could result in insufficient power to detect treatment effects. In addition, the IQ, social interaction style (64, 65), the severity of the disorder (66), and co morbidity (67) in children with ASD may vary widely and these factors may influence the impact of the treatment (68).

**Limitations: **The sample size could be viewed as a potential limitation of this study and the results could be difficult to relate to the population of ASD individuals. Accessibility to one scale for rating the social skills could be another limitation of this study. In conclusion, this study could be expanded to include a larger population of individual with ASD. Further studies resulting from this research could be beneficial for variety of population (individuals with intellectual disabilities, children with hearing impairment etc.).
